# Pregnancy complications and birth outcomes among women experiencing nausea only or nausea and vomiting during pregnancy in the Norwegian Mother and Child Cohort Study

**DOI:** 10.1186/s12884-015-0580-6

**Published:** 2015-06-23

**Authors:** Arthur Chortatos, Margaretha Haugen, Per Ole Iversen, Åse Vikanes, Malin Eberhard-Gran, Elisabeth Krefting Bjelland, Per Magnus, Marit B. Veierød

**Affiliations:** Oslo Centre for Biostatistics and Epidemiology, Institute of Basic Medical Sciences, University of Oslo, PO Box 1122, Blindern, N-0317 Oslo Norway; Department of Nutrition, Institute of Basic Medical Sciences, University of Oslo, PO Box 1046, Blindern, N-0317 Oslo Norway; Division of Environmental Medicine, Norwegian Institute of Public Health, PO Box 4404, Nydalen, N-0403 Oslo Norway; Department of Hematology, Oslo University Hospital, PO Box 4950, Nydalen, N-0424 Oslo Norway; Division of Epidemiology, Norwegian Institute of Public Health, PO Box 4404, Nydalen, N-0403 Oslo Norway; Department of Psychosomatics and Health Behaviour, Norwegian Institute of Public Health, PO Box 4404, Nydalen, N-0403 Oslo Norway; Health Services Research Unit, Akershus University Hospital, PO Box 1000, N-1478 Lørenskog, Norway

**Keywords:** Nausea and vomiting in pregnancy, Pregnancy hormones, Pelvic girdle pain, Pregnancy outcomes, Birth outcomes, MoBa

## Abstract

**Background:**

To compare pregnancy complications and birth outcomes for women experiencing nausea and vomiting in pregnancy, or nausea only, with symptom-free women.

**Methods:**

Pregnancies from the Norwegian Mother and Child Cohort Study (*n* = 51 675), a population-based prospective cohort study, were examined. Data on nausea and/or vomiting during gestation and birth outcomes were collected from three questionnaires answered between gestation weeks 15 and 30, and linked with data from the Medical Birth Registry of Norway. Chi-squared tests, one way analysis of variance, multiple linear and logistic regression analyses were used.

**Results:**

Women with nausea and vomiting (NVP) totalled 17 070 (33 %), while 20 371 (39 %) experienced nausea only (NP), and 14 234 (28 %) were symptom-free (SF). When compared to SF women, NVP and NP women had significantly increased odds for pelvic girdle pain (adjusted odds ratio, aOR, 2.26, 95 % confidence interval, 95 % CI, 2.09–2.43, and aOR 1.90, 95 % CI, 1.76–2.05, respectively) and proteinuria (aOR 1.50, 95 % CI 1.38–1.63, and 1.20, 95 % CI 1.10–1.31, respectively). Women with NVP also had significantly increased odds for high blood pressure (aOR 1.40, 95 % CI 1.17–1.67) and preeclampsia (aOR 1.13, 95 % CI 1.01–1.27). Conversely, the NVP and NP groups had significantly reduced odds for unfavourable birth outcomes such as low birth weight infants (aOR 0.72, 95 % CI 0.60–0.88, and aOR 0.73, 95 % CI 0.60–0.88, respectively) and small for gestational age infants (aOR 0.78, 95 % CI 0.73–0.84, and aOR 0.87, 95 % CI 0.81–0.93, respectively).

**Conclusions:**

We found that women with NVP and NP are more likely to develop pregnancy complications, yet they display mostly favourable delivery and birth outcomes.

**Electronic supplementary material:**

The online version of this article (doi:10.1186/s12884-015-0580-6) contains supplementary material, which is available to authorized users.

## Background

Nausea and vomiting in pregnancy (NVP) or nausea only (NP) have been conditions reportedly accompanying gestation for over 4000 years. Global prevalence varies from approximately 50 to 80 % of all pregnancies [1–5]. The wide variation may be attributable to a lack of universally accepted definitions of these two conditions, which span from slight dizziness and dry retching to continuous vomiting. Hyperemesis gravidarum (HG) is an extreme form of NVP, whereby the extent of vomiting may be so profound that weight loss, electrolyte imbalance, and dehydration requiring hospitalisation result [6]. The NVP condition appears to be so ubiquitous that it has been suggested as being the norm rather than the exception during pregnancy [7]. The aetiology of NVP remains unknown, although it is currently believed to be related to early pregnancy hormones [3, 4].

Lately there has been a resurgence of interest in topics related to NVP, such as diet, gestational conditions and birth outcomes [8–10]. In studies addressing pregnancy complications and birth outcomes for NVP women, results have often been contradictory. Although previous studies found no correlation between NVP and complications such as preeclampsia, diabetes, hypertension, or proteinuria [1, 5, 11], others reported an increased risk of gestational diabetes, hypertension, and preeclampsia for NVP women [8, 12]. Women with NVP were found more likely to have a longer gestation and a consequent lower risk of delivering preterm (< week 37) compared to symptom-free women [5, 13–15], but no association and an increased risk have also been reported [6, 8, 16, 17]. Birth weight reports have also varied in studies of differing design and size, with some observing heavier birth weights from NVP mothers [13, 18, 19], some observing lighter birth weights [9, 20, 21], and some reporting no difference [5, 7, 11, 14–17, 22].

Studies exploring birth anomalies have found no significant differences in the occurrence of these outcomes for women with NVP [7, 16, 23, 24], although a few studies have found a significantly higher number of births with anomalies or malformations for women with HG [20, 25, 26]. Additionally, NVP has been associated with increased numbers of female gender births [6, 8, 15, 17, 25, 27], although some studies found no association between NVP and gender [9, 22, 24], and one study found that NVP was associated with male gender [28].

The Norwegian Mother and Child Cohort study (MoBa) includes a large sample of pregnancies, together with information regarding nausea and vomiting during pregnancy, providing an ideal opportunity to study some of the contradictory results in the literature. In addition, the cohort has been linked with the Medical Birth Registry of Norway (MBRN), allowing access to key birth outcomes.

The aim of this paper was to use the MoBa study to compare pregnancy complications and birth outcomes in full term pregnancies for the women that experience NVP or NP, compared with symptom-free (SF) women.

## Methods

### The Norwegian Mother and Child Cohort Study (MoBa)

MoBa is a prospective population-based pregnancy cohort study conducted by the Norwegian Institute of Public Health [29]. Participants were recruited from all over Norway from 1999 to 2008 and the women consented to participation in 40.6 % of the pregnancies. The women were asked to answer three questionnaires during pregnancy, and follow-up questionnaires were delivered postpartum at regular intervals. Pregnancy and birth records from the MBRN were linked to the MoBa cohort [30]. The present study used the quality-assured data files entitled ‘version 4’ which consisted of 108 842 children.

### Questionnaires and variables

The MoBa data in this study were primarily drawn from the three self-administered questionnaires answered in gestational weeks 15 (Q1), 18–22 (Q2), and 30 (Q3), respectively. Q1 was a general questionnaire covering details regarding maternal health, lifestyle, demographics, previous pregnancies, as well as early reports of nausea and/or vomiting. From Q1 we used maternal height (cm), weight at the start of pregnancy (kg), number of previous pregnancies, previous experiences of NVP (yes or no), previous experiences of pelvic girdle pain (PGP; yes or no), previous preeclampsia (yes or no), diabetic history pre-pregnancy (yes or no), education (seven categories collapsed into: ≤12 years, 13–16 years, or ≥17 years), previous stillbirths or spontaneous miscarriages (yes or no), and smoking during pregnancy (no, sometimes, or daily). Maternal body mass index (BMI, kg/m^2^) was calculated at the start of pregnancy. Parity data from the MBRN and questions detailing previously occurring pregnancies from Q1 were combined in order to minimise missing values [31]. Q2 included a semi-quantitative and validated food frequency questionnaire designed to capture dietary habits during the first 4–5 months of pregnancy, enabling assessment of energy intake (kJ) [32]. Version two of Q2 included detailed questions regarding nausea and vomiting, in addition to the food frequency questionnaire, thus only the women answering version two were included in this study. The nausea and vomiting questions probed experiences of nausea or vomiting during pregnancy (yes or no), the gestational week of onset and cessation, and whether women were still experiencing nausea and/or vomiting at the time of answering the questionnaire.

Data from Q3 enabled us to differentiate NVP cases from women diagnosed with HG, defined as prolonged nausea and vomiting during pregnancy requiring hospitalisation before week 25 of gestation. Q3 also provided data regarding history of high blood pressure prior to pregnancy (yes or no), PGP (defined as self-reported mild or severe pain in the anterior and bilateral posterior pelvis, experiences of ≥3 weeks; yes or no), severe PGP (defined as PGP in addition to the pubic bone region with severe pain reported in all three pelvic locations), high blood pressure in pregnancy (≥3 weeks; yes or no), and proteinuria (≥3 weeks with protein in urine; yes or no). Women answering Q3 also had an option to report their highest recorded systolic and diastolic pressure readings. We included one question, answered 6 months postpartum in questionnaire four (Q4), regarding reasons for caesarean delivery (breech, previous caesarean delivery, pregnancy complication/ill mother, poor growth/fetus complication, own preference, or other).

Data regarding maternal age (year), parity (para 0, para 1, or para ≥2), gestational length (weeks), preeclampsia (defined as eclampsia during birth, during pregnancy and/or post-partum, light or serious preeclampsia, or HELLP syndrome; eleven categories collapsed into yes or no), gestational diabetes (five categories collapsed into yes or no), birth type (five categories collapsed into normal cephalic or other presentations than normal cephalic), caesarean delivery type (no, planned, emergency, or unspecified), placental- and birth weights (g), gender of infant (male or female), body length and head circumference of infant (cm), Apgar scores after 5 min (0–10 score categorised into 0–6 or 7–10), birth defects (yes or no), and infant mortality (eight categories collapsed into born alive (lived >1 y) or born alive then died ≤1 y post-delivery) were obtained from the MBRN. Further details of the diagnostic criteria used in the MBRN for preeclampsia and gestational diabetes can be found elsewhere [33, 34]. Birth defects were defined as any birth defect or malformation registered in the MBRN using the International Classification of Diseases chapter 17 definition [35, 36]. Gestational length calculations were based on ultrasound estimates. The small for gestational age (SGA) variable was created calculating the 10th birth weight percentile of each gestational week from MoBa and MBRN data, based on parity (para 0 vs. para ≥1). Owing to some extreme outlier values for placental- and birth weights, as well as for body length of infant and head circumference in the data set of the MBRN, only values within three standard deviations from the mean for these variables were included in the analyses: placenta weight 99–1258 g, birth weight 1937–5242 g, length 43.1–57.6 cm, and head circumference 30.3–40.3 cm. Outcome questions from either MoBa or the MBRN allowing only for an answer of ‘yes’ had non-answers recoded to ‘no’ or ‘none’.

### Study sample

The present study included 108 842 children, and exclusions were made for multiple births (*n* = 3805), and for women not answering Q1 or only version one of Q2 (*n* = 14 190). We also excluded women not answering version 2 of Q2 (*n* = 5390), or questions relating to episodes of nausea and/or vomiting, or reporting a duration of nausea or vomiting longer than 26 weeks (*n* = 3040). Women recalling NVP symptoms in Q2 contradicting their previous answers reported weeks earlier in Q1 were excluded (*n* = 15 791). Women reporting only vomiting, or hospitalisation due to HG, and women with an energy intake outside the range of 4500–20 000 kJ were also excluded (*n* = 2358). Women with multiple participation in MoBa due to additional pregnancies had all but their first participation excluded (*n* = 9699). Finally, women with a gestational length outside weeks 28–42, women without a full term pregnancy (i.e. non-living births), and those with missing weight and height at the start of pregnancy were excluded (*n* = 2894).

In total, 51 675 women were included in the final study sample: 17 070 (33 %) reporting NVP, 20 371 (39 %) reporting NP, and 14 234 (28 %) SF. This is the same sample used in our previous analysis of NVP in relation to maternal diet and lifestyle, and a detailed description and flow diagram presenting exclusion criteria can be found there [10]. Moreover, severity of symptoms was reported, and the NVP group had a significantly longer mean duration of nausea than the NP group (9.6 weeks versus 7.4 weeks, *p* < 0.001) [10].

Further to these exclusions, women reporting diabetes pre-pregnancy from Q1 (*n* = 251) were excluded from analyses of gestational diabetes, creating a sub-group called ‘gestational diabetes-no prior history’ (*n* = 51 424). Women reporting high blood pressure prior to pregnancy from Q3 (*n* = 1491) were excluded from analyses of high blood pressure, creating a sub-group called ‘high blood pressure-no prior history’ (*n* = 43 089).

### Statistical analyses

The study sample was divided into three groups (SF, NP, or NVP), reflecting answers concerning experiences of nausea and vomiting. Results are presented as means (standard deviations; SDs) or frequencies (%). Chi-squared tests were performed for categorical variables. Outcomes investigated using logistic regression included PGP, severe PGP, high blood pressure-no prior history, proteinuria, preeclampsia, gestational diabetes-no prior history, emergency caesarean delivery, birth type, preterm births (<37 weeks), Apgar scores after 5 min, low birth weight (<2500 g), SGA, birth defects, and gender of infant in relation to group (SF, NP, NVP). Associations between continuous outcomes (birth weight, body length, and head circumference) and group (SF, NP, NVP) were studied by linear regression. Logistic regression models (except low birth weight and gender of infant) included maternal age (continuous), BMI (continuous), smoking during pregnancy, parity, education, and gender of infant. Gender of infant analysis included the same covariates minus gender of infant. Logistic regression of low birth weight and linear regression analysis of birth weight, body length, and head circumference additionally included adjustments for gestational length (continuous) and energy intake (continuous). Gestational diabetes, preeclampsia and high blood pressure-no prior history were also included in the model analysing birth weight and low birth weight outcomes, however their addition gave similar results and were therefore omitted from the presentation of result. Results are presented as crude odds ratios (cOR) and adjusted odds ratios (aOR) or mean differences with 95 % confidence intervals (95 % CIs). Statistical interaction effects were studied and these results are presented in supplementary tables. A significance level of 0.05 was used. All analyses were performed using SPSS 20.0 (IBM Corp, Armonk, NY, USA). We have followed the standard criteria in the Strengthening the Reporting of Observational Studies in Epidemiology (STROBE) statement (http://www.strobe-statement.org).

### Details of ethics approval

The present study was conducted according to the guidelines laid down in the Declaration of Helsinki and all procedures involving human subjects were approved by the Regional Committee for Medical and Health Research Ethics in South-Eastern Norway Committee A (REC South East A, reference numbers S-97045 and S-95113) and the Norwegian Data Protection Authority. Written informed consent was obtained from all MoBa participants.

## Results

### Maternal and gestational history

Maternal demographics and characteristics for the NVP (33 %), NP (39 %), and SF (28 %) groups relevant for the multivariable analyses are shown in Table 1. The total study sample compared with those women excluded from the sample display similar values in regards maternal age, BMI and energy intake. The proportions of para 0 women, women with ≤12 years education and daily smokers during pregnancy are slightly lower in the study sample. However, among the excluded women, 13.2 % (*n* = 5438/41 195) had missing values on education and 14.4 % (*n* = 5922/41 195) had missing values on smoking. The SF group had the lowest proportion of those with high blood pressure pre-pregnancy and the highest proportion of those with diabetes pre-pregnancy (Table 2). Moreover, amongst the para ≥1 women, the SF group had the lowest proportion of reported previous stillbirths or spontaneous miscarriages, lowest proportion of previous experiences of PGP, and the lowest proportion of previous preeclampsia.

**Table 1:** Selected characteristics in relation to group (symptom-free (SF), nausea only (NP), and nausea and vomiting (NVP)), including total sample used and those excluded^a^

	SF	NP	NVP	Total sample	Excluded^b^
	(*n* = 14 234)	(*n* = 20 371)	(*n* = 17 070)	(*n* = 51 675)	(*n* = 41 195)
Maternal age at delivery (y), *n* = 51 675	Mean (SD)	Mean (SD)	Mean (SD)	Mean (SD)	Mean (SD)
	30.3 (4.5)	30.8 (4.4)	29.2 (4.6)	30.1 (4.6) (missing = 0 %)	29.8 (4.8) (missing = 1 %)
BMI at pregnancy start (kg/m^2^), *n* = 51 675	23.9 (4.1)	23.9 (4.1)	24.3 (4.6)	24.0 (4.3) (missing = 0 %)	24.4 (4.4) (missing = 16.9 %)
Energy intake (kJ), *n* = 51 675	9539 (2527.8)	9706.2 (2489.0)	9830.7 (2722.1)	9701.5 (2581.2) (missing = 0 %)	9644.5 (4170.1) (missing = 20.8 %)
	n (%)	n (%)	n (%)	n (%)	n (%)
Parity, *n* = 51 648 Para 0	8947 (62.9)	9475 (46.5)	9070 (53.2)	27 492 (49.8) (missing = 0.1 %)	20 513 (53.2) (missing = 0.7 %)
Maternal education (y), *n* = 51 675 ≤ 12 y	4200 (30.2)	5535 (27.7)	5755 (34.5)	15 490 (30.0) (missing = 2.1 %)	13 402 (32.5) (missing = 13.2 %)
Smoking during pregnancy, *n* = 51 329 Daily	946 (6.7)	805 (4.0)	803 (4.7)	2554 (4.9) (missing = 0.7 %)	2629 (6.4) (missing = 14.4 %)

^a^Excerpt of sample characteristics from Chortatos et al. [10]

^b^Total MoBa sample *n* = 108 842 children, minus 1906 2^nd^ or 3^rd^ order children from multiple pregnancies = 106 936 women. Minus 51 675 women included in sample = 55 261. Excluding 14 066 women with ≥1 participation in MoBa gives 41 195 unique cases

**Table 2:** Maternal and gestational history in relation to group (symptom-free (SF), nausea only (NP), and nausea and vomiting (NVP))

		SF	NP	NVP	
	n	(*n* = 14 234)	(*n* = 20 371)	(*n* = 17 070)	
	51 675	n (%)	n (%)	n (%)	*P* value^a^
High blood pressure pre-pregnancy	44 580				
No	43 089	11 938 (96.9)	17 144 (96.7)	14 007 (96.4)	
Yes	1491	380 (3.1)	581 (3.3)	530 (3.6)	0.03
Diabetes pre-pregnancy:	51 675				
No	51 424	14 138 (99.3)	20 292 (99.6)	16 994 (99.6)	
Yes	251	96 (0.7)	79 (0.4)	76 (0.4)	0.001
Para ≥1 women					
Previous stillbirths or spontaneous miscarriages	24 156				
No	17 246	3829 (72.5)	7697 (70.7)	5720 (71.6)	
Yes	6910	1449 (27.5)	3191 (29.3)	2270 (28.4)	0.045
Previous experience of pelvic girdle pain (PGP)	21 114				
No	15 128	3782 (79.4)	6948 (71.4)	4398 (66.5)	
Yes	5986	983 (20.6)	2788 (28.6)	2215 (33.5)	<0.001
Previous preeclampsia	19 184				
No	17 437	4133 (91.9)	8078 (91.7)	5226 (88.9)	
Yes	1747	362 (8.1)	735 (8.3)	650 (11.1)	<0.001

^a^Chi squared test

### Pregnancy complications

When the NVP and NP groups were compared with the SF group, we found significantly increased odds for PGP (aOR 2.26, 95 % CI 2.09–2.43, and aOR 1.90, 95 % CI 1.76–2.05, respectively) and proteinuria (aOR 1.50, 95 % CI 1.38–1.63, and aOR 1.20, 95 % CI 1.10–1.31, respectively, Table 3). In the analysis of severe PGP, we found a significant interaction between group and maternal smoking during pregnancy (*P* = 0.04), whereby the association between group and severe PGP was strongest among non-smokers (aOR 2.36, 95 % CI 1.90–2.94 for NP, and aOR 3.50, 95 % CI 2.82–4.34 for NVP, Table 3). The NVP women also had significantly increased odds for high blood pressure-no prior history (aOR 1.40, 95 % CI 1.17–1.67) and preeclampsia (aOR 1.13, 95 % CI 1.01–1.27), when compared to the SF group. Conversely, the NP group had significantly reduced odds of developing gestational diabetes-no prior history (aOR 0.76, 95 % CI 0.58–0.98), compared to the SF group. Of the 888 women with high blood pressure-no prior history, *n* = 747 recorded a pressure reading. Of these, the mean was 142/88 mmHg in the NVP group (*n* = 299), 142/87 mmHg in the NP group (*n* = 266), and 144/88 mmHg in the SF group (*n* = 182). Of those answering ‘yes’ to high blood pressure prior to pregnancy, *n* = 550 recorded a pressure reading (mean 149/97 mmHg).

**Table 3:** Odds ratios^a^ of pregnancy complications in relation to group (symptom-free (SF), nausea only (NP), and nausea and vomiting (NVP))

			Crude		Adjusted^d^	
Outcome	Group	n (%)^b^	cOR (95 % CI)^c^	*P* value	aOR (95 % CI)	*P* value
Pelvic girdle pain (PGP)	SF	1101 (9.2)	1.00	<0.001	1.00	<0.001
	NP	2919 (17.3)	2.06 (1.92–2.22)		1.90 (1.76–2.05)	
	NVP	2845 (20.3)	2.51 (2.33–2.71)		2.26 (2.09–2.43)	
Severe pelvic girdle pain^e^						<0.001
Smokers	SF	30 (3.0)	1.00		1.00	
	NP	32 (3.9)	1.33 (0.80–2.21)		1.16 (0.69–1.94)	
	NVP	50 (7.1)	2.50 (1.57–3.98)	<0.001	2.20 (1.36–3.56)	
Non-smokers	SF	105 (1.0)	1.00		1.00	
	NP	390 (2.9)	2.79 (2.24–3.46)		2.36 (1.90–2.94)	
	NVP	483 (4.4)	4.32 (3.50–5.33)		3.50 (2.82–4.34)	
Proteinuria	SF	937 (6.8)	1.00	<0.001	1.00	<0.001
	NP	1616 (8.1)	1.22 (1.12–1.32)		1.20 (1.10–1.31)	
	NVP	1716 (10.4)	1.59 (1.46–1.73)		1.50 (1.38–1.63)	
High blood pressure- no prior history	SF	209 (1.8)	1.00	<0.001	1.00	<0.001
	NP	318 (1.9)	1.06 (0.89–1.27)		1.07 (0.89–1.28)	
	NVP	361 (2.6)	1.49 (1.25–1.76)		1.40 (1.17–1.67)	
Preeclampsia	SF	544 (3.9)	1.00	<0.001	1.00	0.004
	NP	657 (3.3)	0.83 (0.74–0.94)		0.94 (0.84–1.06)	
	NVP	735 (4.4)	1.13 (1.01–1.27)		1.13 (1.01–1.27)	
Gestational diabetes- no prior history	SF	111 (0.8)	1.00	0.08	1.00	0.09
	NP	123 (0.6)	0.77 (0.59–0.99)		0.76 (0.58–0.98)	
	NVP	129 (0.8)	0.97 (0.75–1.25)		0.93 (0.71–1.20)	

^a^OR, odds ratio, logistic regression analysis

^b^Number (%) of women with outcome

^c^CI, confidence interval

^d^Adjusted for age, body mass index, smoking during pregnancy, parity, education, and gender

^e^Significant interaction between severe PGP and smoking status during pregnancy (*P* = 0.04)

### Delivery and birth outcomes

The SF group had the highest proportions delivering preterm or with emergency caesarean delivery, in addition to the highest proportion with a caesarean delivery due to breech presentation or poor growth/fetus complications (Table 4). The SF group had infants born with the lowest mean placental- and birth weights.

**Table 4:** Delivery and birth outcomes in relation to group (symptom-free (SF), nausea only (NP), and nausea and vomiting (NVP))

	n	SF	NP	NVP	*P* value^a^
	51 675	(*n* = 14 234)	(*n* = 20 371)	(*n* = 17 070)	
		n (%)	n (%)	n (%)	
Gestation length	51 675				0.001
Normal range 37–42 weeks	49 181	13 460 (94.6)	19 461 (95.5)	16 260 (95.3)	
Early birth 35–36 weeks	1545	491 (3.4)	553 (2.7)	501 (2.9)	
Very early birth 28–34 weeks	949	283 (2.0)	357 (1.8)	309 (1.8)	
Caesarean delivery	51 675				<0.001
No	44 416	12 104 (85.0)	17 654 (86.7)	14 658 (85.9)	
Planned caesarean delivery	2544	644 (4.5)	1027 (5.0)	873 (5.1)	
Emergency caesarean delivery	4065	1270 (8.9)	1447 (7.1)	1348 (7.9)	
Unspecified	650	216 (1.5)	243 (1.2)	191 (1.1)	
If yes to caesarean delivery, reason					<0.001
Breech presentation	756	283 (29.1)	258 (19.9)	215 (21.0)	
Previous caesarean delivery	447	116 (11.9)	194 (14.9)	137 (13.4)	
Pregnancy complication/ill mother	650	175 (18.0)	262 (20.2)	213 (20.8)	
Poor growth/fetus complication	198	68 (7.0)	83 (6.4)	47 (4.6)	
Own preference	314	76 (7.8)	130 (10.0)	108 (10.5)	
Other	931	255 (26.2)	371 (28.6)	305 (29.8)	
Multiple answers	465				
Mortality	51 675				0.60
Born alive, lived >1 y	51 585	14 206 (99.8)	20 340 (99.8)	17 039 (99.8)	
Born alive, died ≤1 y	90	28 (0.2)	31 (0.2)	31 (0.2)	
Anthropometry		Mean (SD)	Mean (SD)	Mean (SD)	*P* value^b^
Weight of placenta at birth (g)	50 115	661.5 (145.2)	677.9 (145.9)	678.3 (144.9)	<0.001
Weight of infant at birth (g)	51 106	3561.5 (515.1)	3638.5 (506.4)	3605.5 (508.6)	<0.001
Length of infant at birth (cm)	49 180	50.4 (2.1)	50.6 (2.0)	50.4 (2.1)	<0.001
Head circ. of infant at birth (cm)	50 319	35.3 (1.5)	35.4 (1.5)	35.3 (1.5)	<0.001

^a^Chi squared test

^b^One way analysis of variance

The NP group had significantly lower odds of experiencing birth outcomes such as an emergency caesarean delivery (aOR 0.91, 95 % CI 0.84–0.99) or having a preterm birth (aOR 0.86, 95 % CI 0.78–0.96, Table 5) when compared to the SF group. Likewise, the NVP group had significantly lower odds of delivering an infant with a birth type other than normal cephalic. When analysing Apgar score after 5 min, we found a significant interaction between group and infant gender (*P* = 0.001); both the NVP and NP group had significantly lower odds of an Apgar score <7 than the SF group if the infant was male. Both NVP and NP groups had significantly lower odds of delivering low birth weight infants or SGA infants than the SF group (aOR 0.72, 95 % CI 0.60–0.88 and aOR 0.73, 95 % CI 0.60–0.88 for low birth weight, and aOR 0.78, 95 % CI 0.73–0.84 and aOR 0.87, 95 % CI 0.81–0.93 for SGA, respectively, Table 5). No significant association was found between group and birth defects (*p* = 0.15), though an indication of increased odds ratio was found when comparing the NP group with the SF group (aOR 1.10, 95 % CI 1.00–1.22). Both NVP and NP groups had significantly increased odds of having an infant of female gender compared to SF women.

**Table 5:** Odds ratios^a^ of birth outcomes in relation to group (symptom-free (SF), nausea only (NP), and nausea and vomiting (NVP))

			Crude		Adjusted^d^	
Outcome	Group	n (%)^b^	cOR (95 % CI)^c^	*P* value	aOR (95 % CI)	*P* value
Emergency	SF	1220 (9.4)	1.00	<0.001	1.00	0.07
Caesarean delivery Preterm birth	NP	1413 (7.6)	0.79 (0.73–0.86)		0.91 (0.84–0.99)	
	NVP	1302 (8.4)	0.88 (0.81–0.96)		0.97 (0.90–1.06)	
	SF	751 (5.4)	1.00	<0.001	1.00	0.02
	NP	879 (4.4)	0.81 (0.73–0.89)		0.86 (0.78–0.96)	
	NVP	800 (4.8)	0.88 (0.80–0.98)		0.91 (0.82–1.01)	
Birth type (other presentations than normal cephalic)	SF	1372 (10.3)	1.00	<0.001	1.00	0.001
	NP	1780 (9.3)	0.89 (0.82–0.96)		0.97 (0.90–1.04)	
	NVP	1363 (8.5)	0.81 (0.75–0.87)		0.87 (0.80–0.94)	
Apgar score after 5 min (<7)^e^						
Male infants	SF	124 (1.6)	1.00	<0.001	1.00	0.006
	NP	93 (0.9)	0.56 (0.43–0.73)		0.65 (0.49–0.85)	
	NVP	90 (1.1)	0.69 (0.53–0.90)		0.75 (0.57–0.99)	
Female infants	SF	68 (1.1)	1.00	<0.05	1.00	0.03
	NP	120 (1.2)	1.13 (0.84–1.52)		1.26 (0.93–1.70)	
	NVP	74 (0.9)	0.78 (0.56–1.09)		0.85 (0.61–1.19)	
Low birth weight	SF	339 (2.5)	1.00	<0.001	1.00	0.001
	NP	326 (1.7)	0.66 (0.57–0.77)		0.73 (0.60–0.88)^f^	
	NVP	305 (1.9)	0.74 (0.64–0.87)		0.72 (0.60–0.88)^f^	
Small for gestational age	SF	1657 (12.0)	1.00	<0.001	1.00	<0.001
(SGA)	NP	1908 (9.6)	0.78 (0.73–0.84)		0.78 (0.73–0.84)^g^	
	NVP	1694 (10.2)	0.84 (0.78–0.90)		0.87 (0.81–0.93)^g^	
Birth defects	SF	674 (4.9)	1.00	0.24	1.00	0.15
	NP	1022 (5.1)	1.06 (0.96–1.17)		1.10 (1.00–1.22)	
	NVP	791 (4.8)	0.98 (0.88–1.09)		1.03 (0.93–1.15)	
Gender of infant	SF	6212 (45.0)	1.00	<0.001	1.00	<0.001
(female)	NP	9736 (49.0)	1.18 (1.13–1.23)		1.18 (1.13–1.23)^h^	
	NVP	8625 (52.1)	1.33 (1.27–1.39)		1.34 (1.28–1.40)^h^	

^a^OR, odds ratio, logistic regression analysis

^b^Number (%) of women with outcome

^c^CI, confidence interval

^d^Adjusted for age, body mass index (BMI), smoking during pregnancy, parity, education, and gender

^e^Significant interaction between Apgar score after 5 min (<7) and gender of infant (*P* = 0.001)

^f^Adjusted for age, BMI, smoking during pregnancy, parity, education, gender, gestational length, and energy intake

^g^Adjusted for age, BMI, smoking during pregnancy, education, and gender

^h^Adjusted for age, BMI, smoking during pregnancy, parity, and education

In linear regression analyses of birth weight we found that NVP and NP women gave birth to significantly heavier infants than the SF women (*p* < 0.001, Table 6). Likewise, the NP women had significantly longer infants, with significantly larger head circumferences than the infants of the SF group (*p* < 0.001). Several interaction effects were revealed in the analysis of birth weight and head circumference (interaction results can be found in Additional file 1: Table S1).

**Table 6:** Mean difference in birth weight, length, and head circumference for the nausea only (NP) and nausea and vomiting (NVP) groups compared to the symptom-free (SF) group^a^

		Crude		Adjusted^b^	
Outcome	Group	Mean (95 % CI)	*P* value	Mean (95 % CI)	*P* value
Birth weight (g), *n* = 51 106	SF	reference	<0.001	reference	<0.001
	NP	76.9 (66.0–87.9)		38.4 (29.2–47.6)	
	NVP	43.9 (32.6–55.4)		20.9 (11.4–30.5)	
Body length (cm), *n* = 49 180	SF	reference	<0.001	reference	<0.001
	NP	0.20 (0.16–0.25)		0.11 (0.07–0.15)	
	NVP	0.08 (0.03–0.12)		0.07 (0.03–0.11)	
Head circumference (cm),	SF	reference	<0.001	reference	<0.001
*n* = 50 319	NP	0.12 (0.09–0.15)		0.06 (0.04–0.09)	
	NVP	0.05 (0.01–0.08)		0.03 (0.00–0.06)	

^a^Linear regression analysis

^b^Adjusted for age, body mass index, smoking during pregnancy, parity, education, gender, gestational length, and energy intake

## Discussion

In this large cohort we found that women experiencing NVP and NP collectively reported significantly higher odds of developing pregnancy complications such as PGP, severe PGP, high blood pressure, proteinuria, and preeclampsia, than the SF women did. Conversely, the NVP and NP women collectively reported significantly lower odds of unfavourable delivery and birth outcomes, such as an emergency caesarean delivery, a birth presentation other than normal cephalic, preterm births, low Apgar scores after 5 min, low birth weight and SGA infants, compared to SF women. The infants of the NVP and NP women were born heavier, longer, and with a larger head circumference. In addition, the NVP and NP women displayed significantly higher odds of giving birth to female infants.

Owing to our strict delineation between NVP and NP, caution must be exercised when comparing our results with previous studies where women with only vomiting, HG, or with NP and NVP combined have been studied. Unspecific classifications may contribute to the conflicting results from different studies.

That the NVP women had the highest odds of developing high blood pressure and proteinuria in pregnancy supports that they also have the highest odds of developing preeclampsia, since the pathophysiology and diagnosis of preeclampsia includes both maladies [37], and is a finding supported elsewhere [8]. The mean systolic and diastolic pressure reported by the three groups promotes this condition to borderline hypertension by definition (i.e. ≥140/90 mmHg) [37].

A large retrospective study observing birth outcomes for women with hypertension in pregnancy found that these women had increased odds for preterm delivery, low birth weight infants, and infants with a low Apgar score [38]. Although this present study focused on different endpoints, the borderline hypertension present in our NVP and NP groups suggest that NVP may have an alternate impact upon these birth outcomes in regards to the contrasting results for these outcomes found here. The association between NVP and preeclampsia remains enigmatic, although severe vomiting has previously been associated with preeclampsia [12, 39]. Correlations have been reported for preeclampsia with abnormal levels of the hormone human chorionic gonadotropin (hCG), as well as the hormone relaxin in first trimester pregnant women [37, 40–42]. Both hormones feature prominently during the first weeks of pregnancy and act in tandem, appropriately raising suspicion as to their contribution to NVP [42]. Currently, hCG is primarily suspected of being responsible for NVP, as the timing of release correlates with the onset of symptoms [3, 43, 44], although relaxin’s timing is near identical [42]. It is noteworthy that low relaxin levels have been implicated in hypertension and preeclampsia by affecting endothelial vasodilation, and relaxin as a treatment for preeclampsia is currently under research [37, 42, 45].

Relaxin has previously been implicated with PGP, however, its role in PGP is currently inconclusive [46, 47]. Although high levels of relaxin have been suggested as contributing in the underlying mechanisms of PGP [47], when examining the other outcomes in our study, low levels of relaxin seem more plausible for the NVP group here. In addition to the examples mentioned, relaxin is also found to be involved in resetting the osmotic threshold for thirst and antidiuretic hormone release during early pregnancy [48]. A low level of relaxin would thus cause a delay in resetting, accompanied by aberrant water intake patterns, elements that have been suggested in previous studies [10, 49]. By virtue of the higher placental- and birth weights observed, the NVP and NP women have most likely experienced a higher plasma volume expansion than the SF group [50], increasing their susceptibility to a delayed reset. Furthermore, abnormal levels of both relaxin and hCG have been implicated with gastrointestinal motility and gastric dysrhythmias, which are factors suggested to contribute to NVP [1, 4, 13, 44, 51], although with an absence of biomarker data in this study these associations are speculative. The hCG-relaxin dialogue hints at the complex role these hormones have in early pregnancy and future NVP research should consider both hormones when designing studies. We have been unable to find comparable studies identifying a relationship between NVP or NP women and PGP and severe PGP as found here, hence our study raises new questions regarding this relationship, particularly concerning the possible link in aetiologies for the two conditions.

The outcomes in this study where NVP and NP vary in significance suggest that these conditions may have differing physiological effects upon the body during gestation. Vomiting in itself may affect maternal and fetal physiology by modulating stress-related and other hormonal activity, nutrient intake, and dehydration, much as HG does [3, 21, 22, 49]. We found that only the NP group had significantly lower odds of developing gestational diabetes. This finding is in contrast to another study [8], however, this may be due to the unspecific definitions for NP/NVP already mentioned, as well as the validity of the diagnosis from the MBRN records [34]. Although not significant, we observed a trend towards higher odds for birth defects in the NP group, a finding similar to that of other studies observing NP women and those only vomiting [24, 52].

All anthropometric measurements of infants were larger for the NVP or NP group than the SF group, supporting previous studies [13, 18, 19]. The lower proportion of SGA infants and higher proportion of term births for the NVP and NP groups undoubtedly contributes to these outcomes and has been shown elsewhere [15, 18], yet there may well be other pathways contributing to this result [3, 53].

We found the NVP and NP women had higher odds of having a female infant. This finding is supported by the numerous other studies of HG and NVP [6, 8, 17, 25, 27]. The average Norwegian male-female proportion of live births between 1999 and 2008 was 51 % male: 49 % female [54], whereas the NVP group in our study had almost the opposite. It has been suggested that gender of the infant may be affected by such elements as the timing of intercourse, coital rate, or the hormonal milieu at the time of conception [55]. Higher levels of estrogen, estradiol, prolactin and hCG have previously been reported in NVP and HG pregnancies, suggesting causal inference regarding gender and NVP [6, 9, 17, 26, 56], although the issue remains speculative.

### Strengths and limitations

The strengths of this study are the large population-based cohort and the linkage to the MBRN, providing thorough and detailed access to pregnancy and birth outcomes. While exploring areas related to gestation, the cohort has specifically addressed NVP-related issues, thus providing an opportunity to differentiate between NP and NVP combined. Since we have proceeded to investigate the same sample featured in our previous study regarding dietary intakes and NVP [10], this study adds a comprehensive wealth of information regarding many outcomes related to the women with NVP and NP. With such a large cohort size, many significant associations tend to appear, yet the merit of these in the clinical setting are not always relevant. Weaknesses also include the reliance on self-reported data from MoBa questionnaires, particularly for nausea and vomiting. Retrospective evaluation of NVP symptoms has been reported as a possible source of bias [57]. We attempted to address this by excluding cases with inconsistencies in NVP symptoms reported between questionnaires (*n* = 15 791) to have as much confidence as possible in the remaining study sample. When we performed sensitivity analyses with the excluded cases included in the sample, the results were mostly unaffected. Women excluded from the present study tended to have similar ages at delivery, BMI and energy intake, although there appeared to be more women with a lower education as well as a higher parity, findings consistent with non-compliance present in other studies [58–60]. The higher number of daily smokers in the excluded group is most likely a product of the high number of missing values for this variable. Furthermore, it has been presented elsewhere that although prevalence estimates such as smoking are under-estimated in the MoBa cohort when compared with the general Norwegian population, the exposure-outcome associations are valid [61].

Defining and categorising the difference between retching and actual vomiting (defining NP and NVP) may also have affected the results. Endocrine and other diseases may provide a differential diagnoses for hypertension, proteinuria, preeclampsia, gestational diabetes and even nausea and vomiting during pregnancy, however, their occurrence in such a large sample would have negligible effects on the results owing to their low prevalence [4, 62].

## Conclusions

We found that women with NVP and NP collectively had higher odds for pelvic girdle pain, severe pelvic girdle pain, high blood pressure before and during pregnancy, proteinuria, and preeclampsia than the SF group. The NP group had reduced odds of gestational diabetes with no prior history. The NVP and NP groups collectively had a higher proportion of term births, and lower odds for an emergency caesarean delivery, birth type other than normal cephalic, preterm birth, low Apgar score after 5 min, low birth weight and SGA infants than the SF group. The NVP and NP groups also had higher odds of giving birth to a female infant.

To our knowledge, this is the first study finding a relationship between NVP or NP and PGP or severe PGP. Importantly, the higher odds for pregnancy complications during gestation for the NVP and NP women are counterbalanced by reduced odds for unfavourable delivery and birth outcomes.

## Additional file

Additional file 1: Table S1. Interaction effects observed in the linear regression analyses of birth outcomes for the nausea only (NP) and nausea and vomiting (NVP) groups compared to the symptom-free (SF) group.

## Abbreviations

aOR: Adjusted odds ratio
BMI: Body mass index
CI: Confidence interval
cOR: Crude odds ratio
hCG: Human chorionic gonadotropin
HG: Hyperemesis gravidarum
MBRN: Medical birth registry of Norway
mmHG: Millimetre of mercury
MoBa: The Norwegian Mother and Child Cohort Study
NP: Nausea only
NVP: Nausea and vomiting in pregnancy
PGP: Pelvic girdle pain
Q1-4: Questionnaire number
SD: Standard deviations
SF: Symptom-free
SGA: Small for gestational age
