# Erratum: Pregnancy complications and birth outcomes among women experiencing nausea only or nausea and vomiting during pregnancy in the Norwegian Mother and Child Cohort Study

**DOI:** 10.1186/s12884-015-0596-y

**Published:** 2015-08-13

**Authors:** Arthur Chortatos, Margaretha Haugen, Per Ole Iversen, Åse Vikanes, Malin Eberhard-Gran, Elisabeth Krefting Bjelland, Per Magnus, Marit B. Veierød

**Affiliations:** Oslo Centre for Biostatistics and Epidemiology, Institute of Basic Medical Sciences, University of Oslo, Blindern N-0317, Oslo, Norway; Department of Nutrition, Institute of Basic Medical Sciences, University of Oslo, Blindern N-0317, Oslo, Norway; Division of Environmental Medicine, Norwegian Institute of Public Health, Nydalen N-0403, Oslo, Norway; Department of Hematology, Oslo University Hospital, Nydalen N-0424, Oslo, Norway; Division of Epidemiology, Norwegian Institute of Public Health, Nydalen N-0403, Oslo, Norway; Department of Psychosomatics and Health Behaviour, Norwegian Institute of Public Health, Nydalen N-0403, Oslo, Norway; Health Services Research Unit, Akershus University Hospital, Lørenskog, N-1478 Norway

## Erratum

After publication of the original article [1] it came to the publisher’s attention that there were errors in the formatting of Table five. The first Outcome ‘Emergency caesarean delivery’ was incorrectly separated by a line break. The second and third Outcomes ‘Preterm birth’ and ‘Birth type (other presentations than normal cephalic)’ were formatted on the incorrect lines. These errors have been corrected in Table 5 below.

**Table 5:** Odds ratios^a^ of birth outcomes in relation to group (symptom-free (SF), nausea only (NP), and nausea and vomiting (NVP))

			Crude		Adjusted^d^	
Outcome	Group	n (%)^b^	cOR (95 % CI) ^c^	P value	aOR (95 % CI)	P value
Emergency	SF	1220 (9.4)	1.00	<0.001	1.00	0.07
caesarean delivery	NP	1413 (7.6)	0.79 (0.73-0.86)		0.91 (0.84-0.99)	
	NVP	1302 (8.4)	0.88 (0.81-0.96)		0.97 (0.90-1.06)	
Preterm birth	SF	751 (5.4)	1.00	<0.001	1.00	0.02
	NP	879 (4.4)	0.81 (0.73-0.89)		0.86 (0.78-0.96)	
	NVP	800 (4.8)	0.88 (0.80-0.98)		0.91 (0.82-1.01)	
Birth type (other	SF	1372 (10.3)	1.00	<0.001	1.00	0.001
presentations than	NP	1780 (9.3)	0.89 (0.82-0.96)		0.97 (0.90-1.04)	
normal cephalic)	NVP	1363 (8.5)	0.81 (0.75-0.87)		0.87 (0.80-0.94)	
Apgar score after 5 minutes (<7)^e^						
Male infants	SF	124 (1.6)	1.00	<0.001	1.00	0.006
	NP	93 (0.9)	0.56 (0.43-0.73)		0.65 (0.49-0.85)	
	NVP	90 (1.1)	0.69 (0.53-0.90)		0.75 (0.57-0.99)	
Female infants	SF	68 (1.1)	1.00	<0.05	1.00	0.03
	NP	120 (1.2)	1.13 (0.84-1.52)		1.26 (0.93-1.70)	
	NVP	74 (0.9)	0.78 (0.56-1.09)		0.85 (0.61-1.19)	
Low birth weight	SF	339 (2.5)	1.00	<0.001	1.00	0.001
	NP	326 (1.7)	0.66 (0.57-0.77)		0.73 (0.60-0.88)^f^	
	NVP	305 (1.9)	0.74 (0.64-0.87)		0.72 (0.60-0.88)^f^	
Small for gestational age	SF	1657 (12.0)	1.00	<0.001	1.00	<0.001
(SGA)	NP	1908 (9.6)	0.78 (0.73-0.84)		0.78 (0.73-0.84)^g^	
	NVP	1694 (10.2)	0.84 (0.78-0.90)		0.87 (0.81-0.93)^g^	
Birth defects	SF	674 (4.9)	1.00	0.24	1.00	0.15
	NP	1022 (5.1)	1.06 (0.96-1.17)		1.10 (1.00-1.22)	
	NVP	791 (4.8)	0.98 (0.88-1.09)		1.03 (0.93-1.15)	
Gender of infant	SF	6212 (45.0)	1.00	<0.001	1.00	<0.001
(female)	NP	9736 (49.0)	1.18 (1.13-1.23)		1.18 (1.13-1.23)^h^	
	NVP	8625 (52.1)	1.33 (1.27-1.39)		1.34 (1.28-1.40)^h^	

^a^OR, odds ratio, logistic regression analysis

^b^Number (%) of women with outcome

^c^CI, confidence interval

^d^Adjusted for age, body mass index (BMI), smoking during pregnancy, parity, education, and gender

^e^Significant interaction between Apgar score after 5 min (<7) and gender of infant (*P* = 0.001)

^f^Adjusted for age, BMI, smoking during pregnancy, parity, education, gender, gestational length, and energy intake

^g^Adjusted for age, BMI, smoking during pregnancy, education, and gender

^h^Adjusted for age, BMI, smoking during pregnancy, parity, and education
