# Supplementary material for: Pregnancy complications and birth outcomes among women experiencing nausea only or nausea and vomiting during pregnancy in the Norwegian Mother and Child Cohort Study
Source: BMC Pregnancy Childbirth. 2015 Jun 23;15:138. doi: 10.1186/s12884-015-0580-6 (PMC4477493; doi:10.1186/s12884-015-0580-6)
Supplement: Additional file 1: Table S1. — Interaction effects observed in the linear regression analyses of birth outcomes for the nausea only (NP) and nausea and vomiting (NVP) groups compared to the symptom-free (SF) group. [file 12884_2015_580_MOESM1_ESM.doc]

**Table S1.**

**Interaction effects observed in the linear regression analyses of birth outcomes for the nausea only (NP) and nausea and vomiting (NVP) groups compared to the symptom-free (SF) group**

**Birth weight (g), n = 49 687^1^**

|  |  |  | **n** | **Adjusted**^2^ | **P value** |
| --- | --- | --- | --- | --- | --- |
| SF |  |  |  | reference | <0.001 |
| NP |  |  |  | 38.4 (29.2-47.6) |  |
| NVP |  |  |  | 20.9 (11.4-30.5) |  |
|  | **Stratified by** |  |  |  |  |
|  | **Age** |  |  |  |  |
| SF |  | ≤19 - 29 y |  | reference | <0.001 |
| NP |  |  | 7536 | 35.9 (21.9-49.9) |  |
| NVP |  |  | 8631 | 7.6 (-6.1-21.3) |  |
|  |  |  |  |  |  |
| SF |  | ≥30 y |  | reference | <0.001 |
| NP |  |  | 12 115 | 40.7 (28.5-52.9) |  |
| NVP |  |  | 7758 | 33.4 (19.9-46.9) |  |
|  |  |  |  |  | P_interaction_ = 0.02 |
|  | **Education** |  |  |  |  |
| SF |  | ≤12 y |  |  | <0.001 |
| NP |  |  | 5412 | 43.2 (25.6-60.9) |  |
| NVP |  |  | 5614 | 7.3 (-10.3-24.9) |  |
|  |  |  |  |  |  |
| SF |  | ≥13 y |  |  | <0.001 |
| NP |  |  | 14 239 | 36.8 (26.1-47.6) |  |
| NVP |  |  | 10 775 | 27.3 (15.9-38.7) |  |
|  |  |  |  |  | P_interaction_ = 0.03 |
|  | **BMI** |  |  |  |  |
| SF |  | ≤18.5 |  | reference | 0.004 |
| NP |  |  | 523 | 83.1 (33.7-132.5) |  |
| NVP |  |  | 535 | 38.2 (-11.5-87.8) |  |
|  |  |  |  |  |  |
| SF |  | 18.5-24.9 |  | reference | <0.001 |
| NP |  |  | 13371 | 31.0 (20.2-41.7) |  |
| NVP |  |  | 10267 | 23.3 (11.8-34.7) |  |
|  |  |  |  |  |  |
| SF |  | ≥25 |  | reference | <0.001 |
| NP |  |  | 5587 | 52.2 (34.1-70.4) |  |
| NVP |  |  | 5757 | 19.2 (0.8-37.5) |  |
|  |  |  |  |  | P_interaction_ = 0.001 |

^1^Birth weight analyses only include infants with weight ≥1937 g and ≤5242 g

^2^Adjusted for age, body mass index (BMI), smoking during pregnancy, parity, education, gender of child, gestational length, energy intake

**Head Circumference (cm)^1^, n** = 48 920

|  |  |  | **n** | **Adjusted**^2^ | **P value** |
| --- | --- | --- | --- | --- | --- |
| SF |  |  |  | reference | <0.001 |
| NP |  |  |  | 0.06 (0.04-0.09) |  |
| NVP |  |  |  | 0.03 (0.00-0.06) |  |
|  | **Stratified by** |  |  |  |  |
|  | **BMI** |  |  |  |  |
| SF |  | ≤18.5 |  | reference | 0.01 |
| NP |  |  | 509 | 0.22 (0.06-0.38) |  |
| NVP |  |  | 527 | 0.22 (0.05-0.38) |  |
|  |  |  |  |  |  |
| SF |  | 18.5-24.9 |  | reference | 0.06 |
| NP |  |  | 13 183 | 0.04 (0.01-0.08) |  |
| NVP |  |  | 10 115 | 0.02 (-0.02-0.06) |  |
|  |  |  |  |  |  |
| SF |  | ≥25 |  | reference | 0.001 |
| NP |  |  | 5673 | 0.10 (0.05-0.16) |  |
| NVP |  |  | 5497 | 0.05 (-0.01-0.10) |  |
|  |  |  |  |  | P_interaction_= 0.001 |
|  | **Parity** |  |  |  |  |
| SF |  | Para 0 |  | reference | 0.34 |
| NP |  |  | 8946 | 0.03 (-0.01-0.07) |  |
| NVP |  |  | 8556 | 0.02 (-0.02-0.06) |  |
|  |  |  |  |  |  |
| SF |  | Para 1 |  | reference | <0.001 |
| NP |  |  | 6596 | 0.12 (0.07-0.18) |  |
| NVP |  |  | 5068 | 0.07 (0.01-0.12) |  |
|  |  |  |  |  |  |
| SF |  | Para ≥2 |  | reference | 0.14 |
| NP |  |  | 3823 | 0.07 (0.00-0.14) |  |
| NVP |  |  | 2515 | 0.05 (-0.03-0.12) |  |
|  |  |  |  |  | P_interaction_ = 0.03 |
|  | **Education** |  |  |  |  |
| SF |  | ≤12 y |  | reference | <0.001 |
| NP |  |  | 5343 | 0.11 (0.06-0.17) |  |
| NVP |  |  | 5513 | 0.04 (-0.01-0.10) |  |
|  |  |  |  |  |  |
| SF |  | ≥13 y |  | reference | 0.03 |
| NP |  |  | 14 022 | 0.05 (0.01-0.08) |  |
| NVP |  |  | 10 626 | 0.03 (-0.01-0.07) |  |
|  |  |  |  |  | P_interaction_ = 0.02 |
|  | **Gestational length** |  |  |  |  |
| SF |  | 37-42 wks |  | reference | <0.001 |
| NP |  |  | 18 697 | 0.06 (0.03-0.09) |  |
| NVP |  |  | 15 525 | 0.03 (-0.01-0.06) |  |
|  |  |  |  |  |  |
| SF |  | 35-36 wks |  | reference | 0.40 |
| NP |  |  | 501 | 0.12 (-0.06-0.30) |  |
| NVP |  |  | 465 | 0.09 (-0.10-0.27) |  |
|  |  |  |  |  |  |
| SF |  | 28-34 wks |  | reference | 0.70 |
| NP |  |  | 167 | -0.03 (-0.37-0.31) |  |
| NVP |  |  | 149 | -0.14 (-0.49-0.21) |  |
|  |  |  |  |  | P_interaction_= 0.02 |

^1^Head circumference analyses only include infants with circumference ≥30.3 cm and ≤40.3 cm

^2^Adjusted for age, BMI, smoking during pregnancy, parity, education, gender of child, gestational length, energy intake
